# Efficacy of the CALM^®^ Algorithm in Reducing Motion-Induced Artifacts in CBCT Imaging: A Fractal Dimension Analysis of Trabecular Bone

**DOI:** 10.3390/dj12080262

**Published:** 2024-08-19

**Authors:** Yahia H. Khubrani, Hassem Geha, Rujuta A. Katkar, Taraneh Maghsoodi-Zahedi, William Moore, Ahmed Z. Abdelkarim

**Affiliations:** 1 Department of Oral and Maxillofacial Radiology, Jazan University College of Dentistry, Jazan 82621, Saudi Arabia; yhkhubrani@jazanu.edu.sa; 2 Department of Comprehensive Dentistry, University of Texas Health San Antonio, San Antonio, TX 78229, USA; geha@uthscsa.edu (H.G.); katkarr@uthscsa.edu (R.A.K.); maghsoodi@uthscsa.edu (T.M.-Z.); moorew@uthscsa.edu (W.M.); 3 Division of Oral and Maxillofacial Radiology, College of Dentistry, The Ohio State University, Columbus, OH 43210, USA

**Keywords:** fractal dimension, cone beam CT, motion, motion artifact reduction algorithms

## Abstract

Objective: The primary goal of this investigation was to ascertain the efficacy of the CALM^®^ motion artifact reduction algorithm in diminishing motion-induced blurriness in Cone Beam Computed Tomography [CBCT] images. The assessment was conducted through Fractal Dimension [FD] analysis of the trabecular bone. Methods and Materials: A desiccated human mandible was subjected to Planmeca ProMax 3D^®^ scanning under eight distinct protocols, marked by variations in motion presence [at 5, 10, and 15 degrees] and the deployment of CALM^®^. In every scan, five distinct regions of interest [ROIs] were designated for FD analysis, meticulously avoiding tooth roots or cortical bone. The FD was computed employing the box-counting method with Image-J 1.53 software. Results: Our findings reveal that a 5-degree motion does not significantly disrupt FD analysis, while a 10-degree motion and beyond exhibit statistical differences and volatility among the sites and groups. A decreased FD value, signifying a less intricate or “rough” bone structure, correlated with amplified motion blurriness. The utilization of CALM^®^ software seemed to counteract this effect in some instances, reconciling FD values to those akin to the control groups. Nonetheless, CALM^®^’s efficacy differed across sites and motion degrees. Interestingly, at one site, CALM^®^ application in the absence of motion resulted in FD values considerably higher than all other groups. Conclusion: The study indicates that motion, particularly at 10 degrees or more, can considerably impact the FD analysis of trabecular bone in CBCT images. In some situations, the CALM^®^ motion artifact reduction algorithm can alleviate this impact, though its effectiveness fluctuates depending on the site and degree of motion. This underscores the necessity of factoring in motion and the employment of artifact reduction algorithms during the interpretation of FD analysis outcomes in CBCT imaging. More research is necessary to refine the application of such algorithms and to comprehend their influence on different sites under varying motion degrees.

## 1. Introduction

Bone strength is closely tied to complex bone architecture [1]. Fractal Dimension analysis [FD] is a mathematical method employed to measure bone quality by quantifying complex bone morphology and irregular structures, often used in the assessment of conditions like osteoporosis [2,3]. White and Rudolph [3] demonstrated a significant difference in trabecular bone patterns between patients with osteoporosis and healthy individuals. FD decreases with lower bone density and increases with higher density, as observed through dual-energy X-ray absorptiometry [4].

Nevertheless, FD’s effectiveness as a predictor of osteoporosis using Cone Beam Computed Tomography [CBCT] is disputed due to inconsistencies and low accuracy in some studies [2,5,6]. However, it is crucial in assessing initial implant stability and the subsequent success of prosthetic treatments [7,8]. Comparisons of FD analysis on CBCT and digital panoramic radiographs show no significant differences [9]. CBCT’s popularity has risen for its wide range of applications, including dento-maxillofacial structure assessments and prosthetic planning [10,11,12], and for examining trabecular changes in Temporomandibular joints [TMJs] [13].

Despite these advancements, the impact of exposure parameters and artifacts on FD analysis through CBCT remains a concern. While variations in kV and mAs do not significantly affect the results, voxel size variations can affect trabecular structure analysis [14]. Motion artifacts, common in children or people with unstable conditions like Parkinson’s disease, can complicate interpretation [15,16]. Despite these challenges, FD’s use in maxillofacial conditions remains widely studied [1,2,3,5,6,13,17,18]. Motion artifacts can significantly affect the interpretation of the images, leading to the development of motion artifact reduction algorithms like CALM^®^ in Planmeca Promax 3D [15].

The effectiveness of such algorithms in restoring fine details, such as trabecular bone patterns, is crucial for accurate FD analysis. However, to our knowledge, no studies have yet quantitively evaluated the effectiveness of motion artifact reduction algorithms like CALM^®^ in reducing motion unsharpness as measured through FD analysis.

Therefore, this study aimed to evaluate the effectiveness of CALM^®^ in reducing motion unsharpness as measured through fractal analysis. We hypothesized that the application of CALM^®^ would significantly reduce motion unsharpness, thereby improving the accuracy of FD analysis of the trabecular bone using CBCT images.

Our null hypothesis is that there is no significant difference in the FD analysis of CBCT images with and without the application of CALM^®^. Therefore, different degrees of motion artifacts at 5, 10, and 15 degrees should not affect FD analysis on CBCT images after the application of CALM^®^ software.

## 2. Materials and Methods

**Figure 1:**
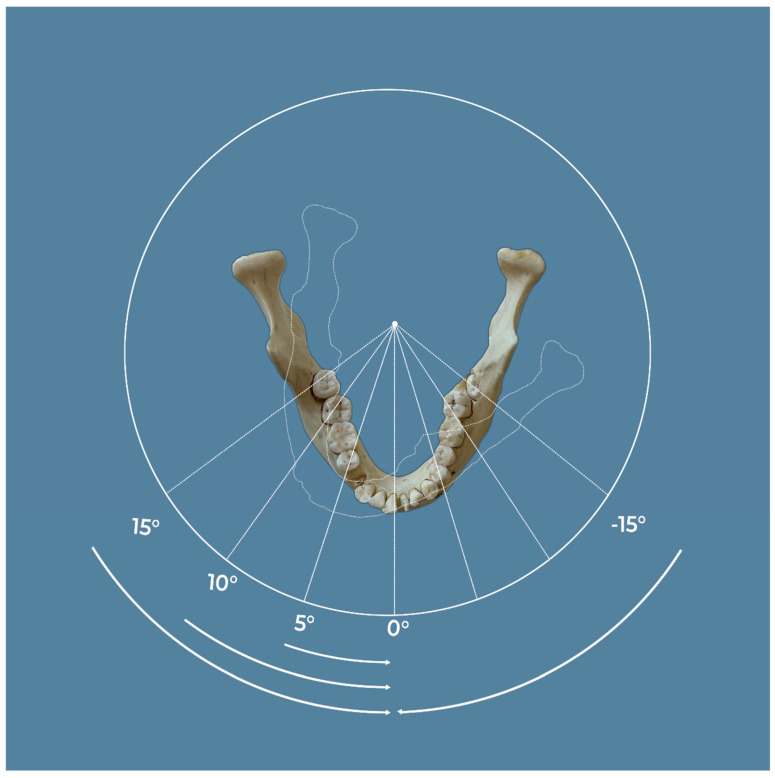
A dry human mandible was scanned using the Planmeca ProMax 3D^®^ system with a 10 × 6 cm field of view [FOV], operating at 90 kV, 10 mA, for 15 s, and a voxel size of 150 µm [high definition]. The scanning protocols were: Group 1 with no motion and no CALM^®^, Group 2 with no motion but with CALM^®^, Group 3 with 5° motion and no CALM^®^, Group 4 with 5° motion and CALM^®^, and Group 5 with 10° motion and no CALM^®^.

A dry human mandible (Figure 1) was scanned using the Planmeca ProMax 3D^®^ system (Planmeca, Helsinki, Finland), which employed a field of view [FOV] of 10 × 6 cm, set at 90 kV and 10 mA, with an exposure duration of 15 s. The scans achieved a high-definition resolution with a voxel size of 150 µm. Eight distinct scanning protocols were tested to evaluate the influence of movement and the use of the Correction Algorithm for Latent Movement [CALM^®^]. The first group was scanned without any motion or CALM^®^. In the second group, scans were conducted without motion but with CALM^®^ activated. For the third and fourth groups, a 5-degree motion was introduced, with the third group scanned without CALM^®^ and the fourth with CALM^®^. The fifth and sixth groups involved a 10-degree motion, with the fifth group lacking CALM^®^ and the sixth utilizing CALM^®^. Lastly, the seventh and eighth groups underwent a 15-degree motion, with the seventh group scanned without CALM^®^ and the eighth group with CALM^®^. This comprehensive setup enabled a thorough investigation into the effects of motion and the application of CALM^®^ on the image quality.

All motions were induced clockwise with a rotating device using a remote control and returned to the initial position. One person induced the motion to ensure accuracy and reproducibility. To control for variability in motion introduction, we manually controlled the motion using a remote control. We acknowledge the potential variability introduced by this method and suggest that future studies employ automated motion control systems to enhance reproducibility and reduce variability.

A pilot study was carried out using identical parameters, involving five scans per group and three site measurements. Based on the pilot study’s results with 15 samples per group, mean and standard deviations were calculated. Power analysis, conducted using GPower software version 3.1.2 (Heinrich-Heine-Universität Düsseldorf, Düsseldorf, Germany), yielded an effect size of 0.2976 with an alpha error of 0.05, and which required 90% power. This necessitated a total sample size of 216 distributed across 8 groups, resulting in 27 per subgroup. Thus, 80 scans, 10 per group, were obtained.

To control variables, all exposure parameters and mandible positions were fixed. The mandible was secured during image acquisition to limit motion. The final 80 scans, excluding the pilot, were captured in a single day, maintaining uniform positioning and slice selection. For the fractal analysis, five regions of interest [ROIs] were selected in each scan. These regions were the right molar [site #1] and premolar [site #2] regions, anterior [site #3] region, and left premolar [site #4] and molar [site #5] regions. Each ROI was a square of 128 × 128 pixels, chosen to avoid tooth roots or cortical bone. The ROIs were selected by a trained dental radiologist with over ten years of experience in CBCT imaging and fractal analysis.

**Figure 2:**
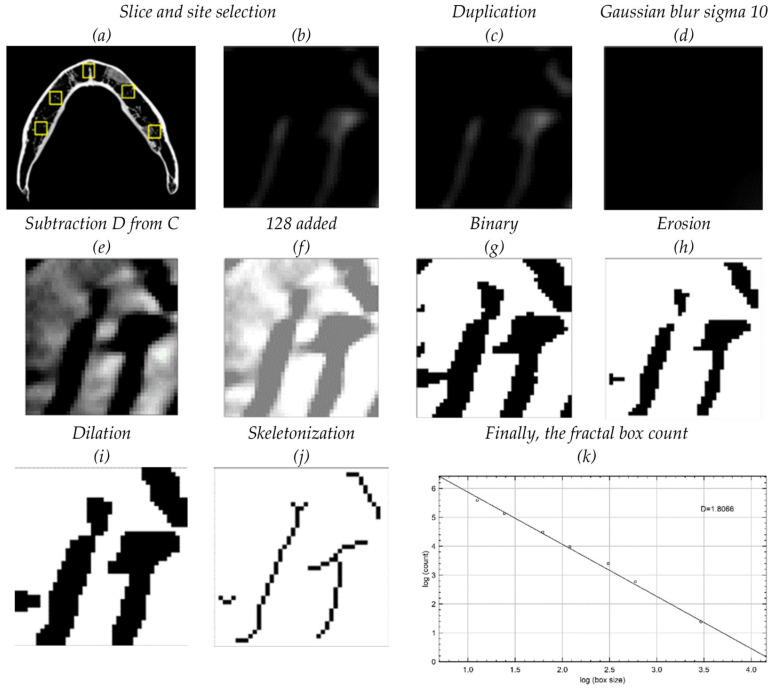
Image analysis: One slice [#210] was selected apical to the root apices. Five areas of interest were chosen: right and left premolars, right and left molars, and anterior. (**a**,**b**) Region of interest [ROI] with the same axial slice number is used for reproducibility purposes. (**c**,**d**) The cropped ROI was duplicated (**c**) and then blurred with a Gaussian filter (**d**). (**e**,**f**) The blurred image was subtracted from the original image (**e**), and 128 was added to the result at each pixel location (**f**). (**g**) The resultant image was converted to binary, to set the image into trabeculae and marrow spaces. (**h**,**i**) The binary image was eroded and then dilated to reduce the noise before skeletonization. (**j**) The skeletonized image, which corresponds to trabeculae, was used for fractal analysis. A single ImageJ macro was used for all measurements to reproduce exact locations and measurements.

The fractal dimension [FD] was calculated using the box-counting method. This method involves overlaying the image with a grid of boxes and counting the number of boxes that contain part of the image. This process is repeated with different box sizes, and the FD is calculated as the slope of the line when plotting the logarithm of the box size against the logarithm of the box count (Figure 2). This calculation was performed using Image-J 1.53 software (National Institutes of Health, Bethesda, MD, USA), following the method described by Magat et al., 2022 [9].

**Figure 3:**
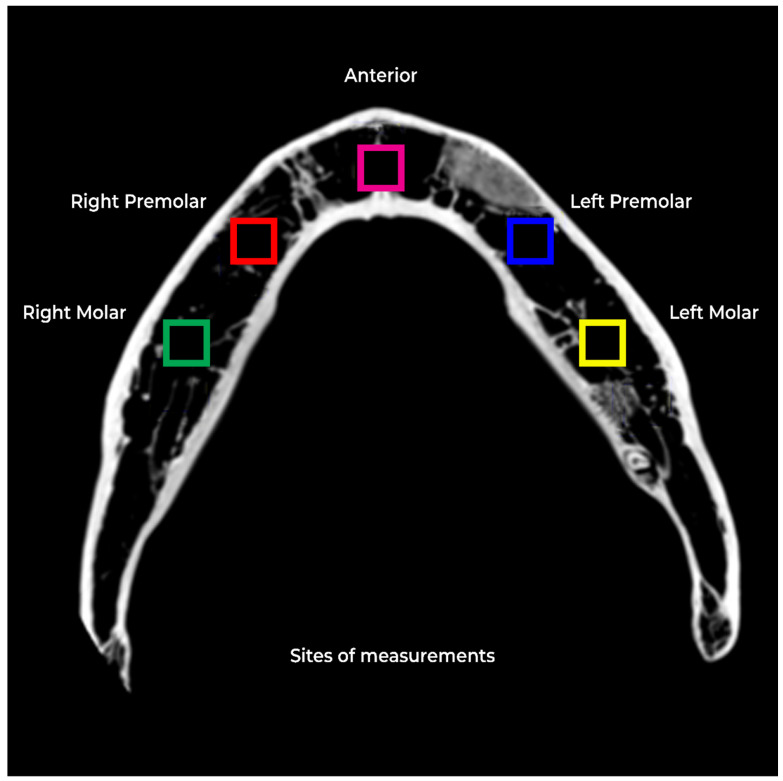
Five site measurements were performed for each group.

In the fractal analysis, five site measurements were performed for each group (Figure 3). The mean, standard deviation, and standard error were calculated, and a 95% confidence interval was used. A One-Way ANOVA with Post-Hoc Tukey HSD Test was used for intergroup comparisons for each site. Groups 1 and 2 were used as a baseline, to which all other groups were compared.

## 3. Results

**Figure 4:**
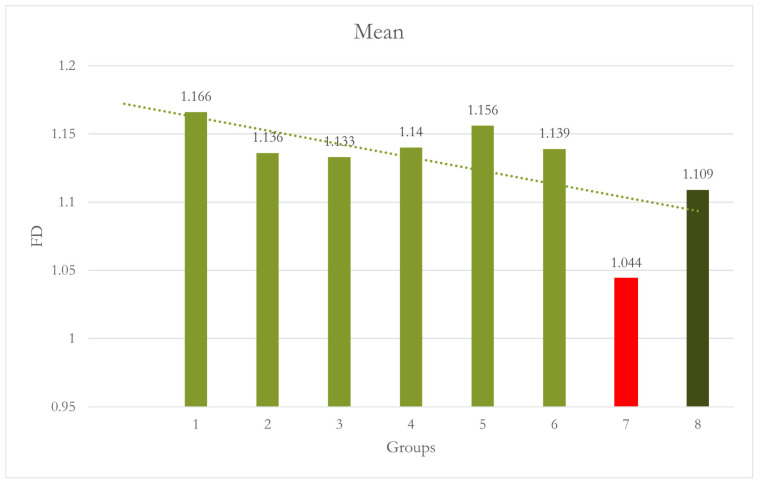
Graph shows Group 7 [15º No CALM^®^]: motion caused significantly lower FD values than Group 1 [*p* = 0.005].

At Site 1 [Right Molar (RM)], the difference between Group 7 [15D no CALM^®^] and Group 1 was statistically significant [*p* = 0.005]. Group 7 shows significantly lower FD values. All other groups were not significantly different when compared to group 1 as well as Group 2. CALM^®^ appears to have restored the values of FD [Table 1 and Figure 4].

**Figure 5:**
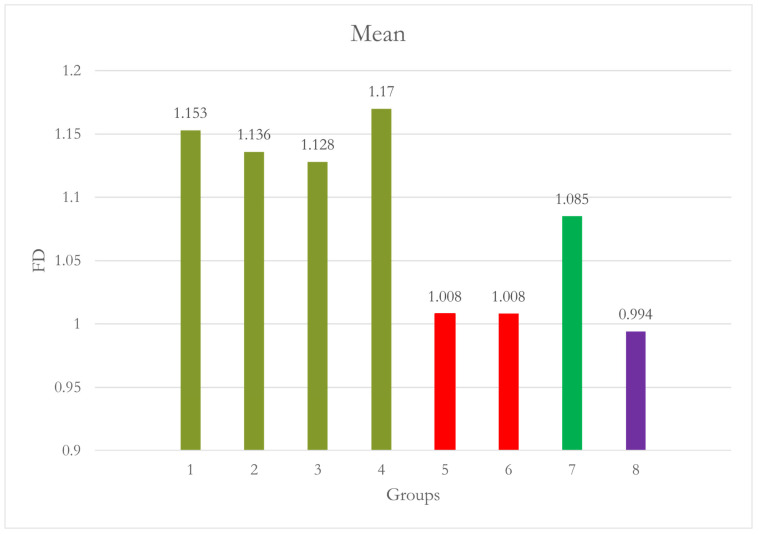
Group 5 [10° No CALM^®^] and Group 6 [10° with CALM^®^] 10° motion with or without CALM^®^ caused significantly lower FD values than Groups 1 and 2. A 15° motion in Group 7 did not significantly affect FD. However, Group 8 [15° with CALM^®^] had significantly lower FD values than Groups 1 and 2.

At Site 2 [RPM], Group 5, Group 6, and Group 8 exhibited significantly lowered FD values compared to both Groups 1 [*p* = 0.002, *p* = 0.002, and *p* < 0.001, respectively] and Group 2 [*p* = 0.009, *p* = 0.009, and *p* = 0.002, respectively]. The 15° motion in Group 7 did not significantly affect FD. However, Group 8 [15° with CALM^®^] had significantly lower FD values than Groups 1 and 2 [Table 2 and Figure 5].

**Figure 6:**
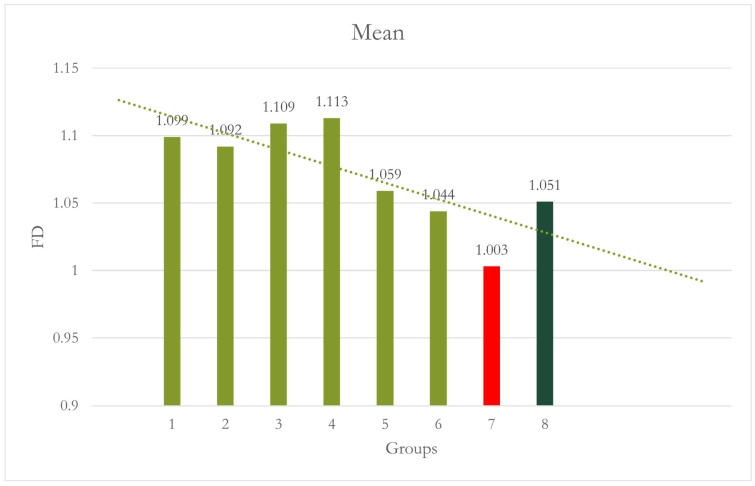
Group 7 [15° No CALM^®^]: motion caused significantly lower FD values than Groups 1 and 2. Group 8 [15° with CALM^®^]: CALM^®^ restored the values of FD.

At Site 3 [anterior], the difference between Group 1 and Group 7 was statistically highly significant [*p* < 0.001], and Group 7 and Group 2 were statistically significant [*p* = 0.001]. Group 7 showed significantly lowered FD values. CALM^®^ seems to have restored the values of FD values in Group 8 [Table 3 and Figure 6].

**Figure 7:**
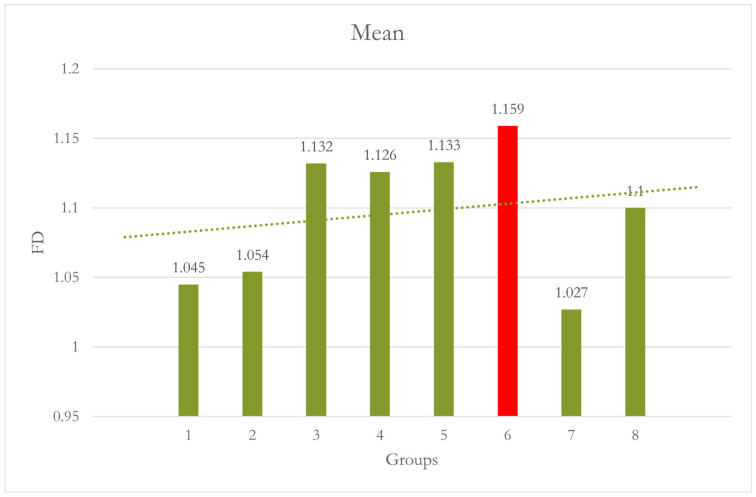
Group 7 [15° No CALM^®^]: motion caused significantly lower FD values than Groups 1 and 2. Group 8 [15° with CALM^®^]: CALM^®^ restored the values of FD.

At Site 4 [Left premolar (LPM)], Group 6 showed a significant difference when compared to both Group 1 [*p* = 0.007] and Group 2 [*p* = 0.017]. Combining motion and CALM^®^ caused significantly higher FD values than Groups 1 and 2. Although the values of Groups 3, 4, 5, and 8 are also higher than Groups 1 and 2, they are not statistically significant [Table 4 and Figure 7].

**Figure 8:**
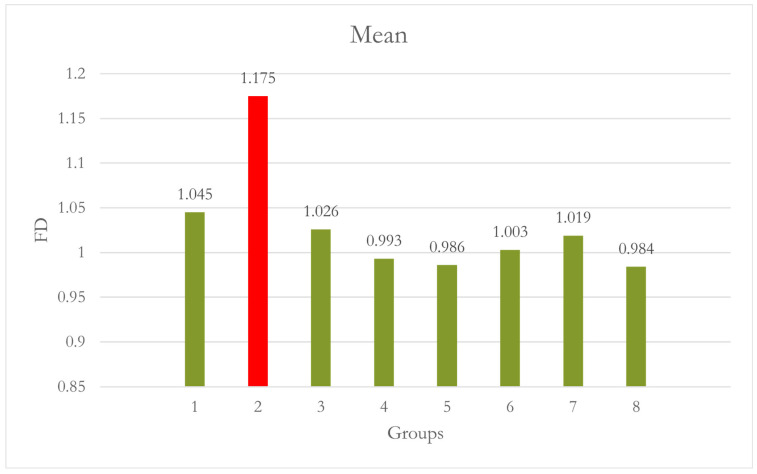
Group 2 [0° with CALM^®^]: using CALM^®^ alone caused significantly higher FD values than all other groups. Motion did not significantly affect the FD at this site, although it seems to have caused a slight decrease in FD values.

**Figure 9:**
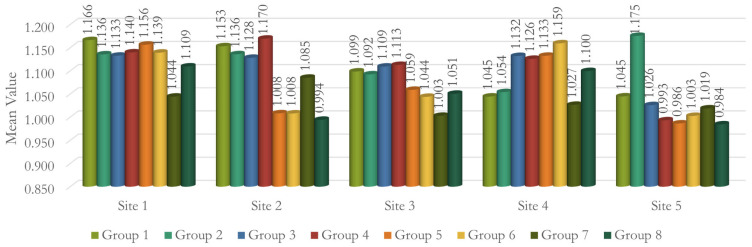
Graph shows a summary of the results of all groups.

At Site 5 [LM], Group 1 and Group 2 were significantly different from each other [*p* = 0.040], while all other groups were not significantly different from Group 1. When compared with Group 2, all the groups showed significant differences, with Group 5 and Group 8 showing highly significant differences [*p* < 0.001]. Using CALM^®^ alone in Group 2 caused significantly higher FD values than all the other groups [Table 5 and Figure 8]. A summary of all the groups’ results is shown in Figure 9.

The results show that a 5-degree motion did not significantly affect the FD analysis, while 10-degree motion and higher showed statistical differences and variability between the sites and groups. A lower FD value, indicating less complexity or “roughness” in the bone structure, was associated with greater motion unsharpness. The application of CALM^®^ software appeared to mitigate this effect in some cases, restoring FD values closer to those of the baseline groups.

However, the effectiveness of CALM^®^ varied between sites and degrees of motion. Notably, at Site 5, the application of CALM^®^ in Group 2 [no motion] resulted in significantly higher FD values than all other groups, suggesting that CALM^®^ may affect the measurements even in the absence of motion.

Additional scans were acquired to further investigate these results. These scans confirmed that the application of CALM^®^ could exaggerate the effect of motion, leading to significantly lower FD values, particularly in the presence of a 15-degree counterclockwise motion.

These additional measurement results in Table 6 show that Group 5 has a significantly lower value than Group 1 [*p* = 0.005] and Group 2 [*p* = 0.008]. The results show that CALM^®^ in Group 2 without motion produces no significant effect. A 15° motion in Group 3 reduced FD, but the values were not statistically significant. CALM^®^ in Group 4 exaggerated the effect of motion and produced significant results. Group 1, Group 2, and Group 3 were not significantly different. Also, the difference between Groups 3 and 4 was not statistically significant. These results agree with the previous findings at Sites 1, 2, and 3, except the scan used for these measurements used a counterclockwise motion direction.

## 4. Discussion

This study aimed to evaluate the effectiveness of the CALM^®^ motion artifact reduction algorithm in reducing motion unsharpness in CBCT images, as measured through fractal dimension [FD] analysis. Several factors were considered as potential causes of those variations. The first is the sensitivity of fractal dimension analysis. Normal FD for healthy bone ranges from 1.1 to 2.68 [9,19,20,21]. According to Magat et al. [9], the variation was mainly due to fractal analysis [FA] rather than the result of different materials, strategies, or anatomic sites used for the studies.

The second factor is the variation in density and grayscale values of the dry mandible used in the study. Perrotti et al. [22] stated that “The more the bone was compact, the higher were FD values”. Additionally, “The increase in the values of the FD strongly correlated with the increase of the percentage of the bone trabeculae observed in the histological slides” [22]. Hua et al. [4] also showed a significant drop in FD with decreased bone density measured on dual-energy X-ray absorptiometry. Both studies indicate that any slight change in including or excluding trabecular bone during FD analysis may change the results.

Southard et al. [19] found a direct relationship between the FD measurements and bone density. Hence, increasing the trabecular bone density translates to higher FD values. However, this study was performed on plain radiographs that were digitized. Therefore, the effect of some parameters, such as voxel size, could not be appreciated. However, other studies showed no correlation between grayscale values and FD [9,17].

Pauwels et al. [14] studied the effect of exposure parameters on bone structure analysis. The study included combinations of kV, mAs, and voxel sizes. Their study concluded that kV above 90 shows significant results with only bone volume per total volume [BV/TV], which is used to assess bone strength by analyzing bone microstructure. Additionally, kV did not affect other trabecular bone analyses, such as FD. In comparison, voxel size was the major factor and significantly affected trabecular bone analysis. The results show a decrease in the FD at larger voxel sizes, equal to or higher than 160 µm. The current study keeps the voxel size to 150 µm [HD] to provide optimal resolution and acceptable noise levels. This may partially undermine the clinical effect of noise from soft tissue.

Some studies revealed that moderate variants in noise level, either due to soft tissue emulators or water, did not significantly impact bone structure parameters on cone beam CT [14,23,24,25]. These studies also elaborated on the variability in the clinical images where the motion artifacts might cause blurring or unsharpness.

Several studies [14,24,26] concluded that kV did not significantly affect bone structural analysis, except for BV/TV values. On the other hand, voxel sizes significantly affect bone structural analysis, such as FD. This is because fine details are lost at larger voxel sizes due to lower spatial resolution [14,27,28].

Therefore, we fixed all previous parameters and focused on the effect of motion on FD. We found that motion and motion correction software affect FD. Additionally, this effect is partially unpredictable, at least in our experiment. Therefore, it is crucial to consider all parameters affecting FD before implementing clinical applications.

The third factor is the direction and timing of motion relative to the scan acquisition. The motion degrees and time of induction were controlled by the researcher using a remote control. This manual control may have resulted in the variability of the motion effect. Additionally, except for Site 5, only a clockwise direction was used in this study. These factors can be better controlled in future studies using a fully automated system.

The fourth factor is CALM^®^. Rigid body movements, such as translation and rotation, are the most common movement in the head and neck [15]. According to Hernandez et al. [15], there are two common ways to detect and correct motion: using head tracking devices or using the motion artifacts metric [MAM] optimization algorithm. CALM^®^, [proprietary for Planmeca Promax 3D^®^], seems to be of the MAM type. It does not need extra head devices and can be applied before or after scanning with a push of a button. In general, the MAM algorithm works on enhancing image sharpness with regularization terms that estimate the motion during reconstruction. This algorithm corrects sharpness, while the trabecular bone pattern has fine details that require high resolution.

Our study showed that CALM^®^ affected FD analysis. It restored the FD of Group 8 at Sites 1 and 2. It failed at Site 2 in Groups 6 and 8. It exaggerated the motion effect at Site 2 in Group 8 and Site 5 in Group 4. It significantly increased the FD in Group 2 at Site 5.

In this study, we used an image-J macro to precisely reproduce the measurements. This macro does not correct for motion. If there is slight inclusion or exclusion in the trabecular bone, it may affect the measurements. This factor is also complicated by the fact that FA itself is variable and technique sensitive [9]. Furthermore, a significant correlation between the changes in bone density and FD values has been documented in the literature [22,23]. All these factors complicate FD measurements and result in variable values.

Several studies have attempted to correlate trabecular bone patterns with osseous diseases/conditions. White and Rudolph [3] were among the first authors correlating FD with altered bone trabeculae in osteoporotic subjects. By contrast, Sindeaux et al. [29] found a correlation between osteoporosis and FD of the cortex of the mandible, not the trabecular bone FD. Alman et al. [30] found that FD can be possibly utilized as a discriminator for people with low bone mineral density on dental radiographs. However, there is documented literature opposing using FD as a screening or adjunctive tool to refer or diagnose patients with osteoporosis [5]. A systematic review and meta-analysis by Franciotti et al. [31] demonstrated heterogeneity in the literature and low reliability in using FD for osteoporosis identification.

Recent studies [32,33] showed that FD could be a useful descriptor for medication-induced osteonecrosis of the jaw [MRONJ]. This could help in the assessment of the disease. Kato et al. [34] assessed the complexity of fibrous dysplasia and ossifying fibroma on CBCT using FD. They discovered fibrous dysplasia might have a significantly more complex structure, represented by higher FD values, than ossifying fibroma.

In general, our research showed that motion beyond 5 degrees led to significant variations in fractal dimension [FD] results, based on site and degree of motion, and CALM^®^ use. The CALM^®^ software generally mitigated motion unsharpness, but its effectiveness varied. At Site 5, CALM^®^ notably increased FD values in a no-motion scenario, suggesting potential effects on measurements without motion.

Despite statistically significant FD differences under various motion conditions, and with or without CALM^®^, the clinical significance of these findings may depend on the specific context and how motion unsharpness affects CBCT image interpretation.

More research is needed to optimize artifact reduction algorithms like CALM^®^ and to comprehend their impact under different motion scenarios. Further studies could also leverage automated systems for more consistent motion control.

## 5. Limitations

This study has several limitations that should be considered when interpreting the results. One of the primary limitations is the manual control of motion using a remote control, which could have introduced variability in the motion effect. This may limit the precision of the results and does not fully represent the range of motion that could occur in a real-world clinical setting. Future studies could benefit from using a fully automated system to control motion more consistently and explore the effects of motion in different directions. Lastly, the study primarily used a clockwise direction of motion, which may not fully represent the range of motion that could occur in a real-world clinical setting.

## 6. Conclusions

The study findings suggest that motion, particularly at 10 degrees and higher, can significantly affect the fractal dimension analysis of trabecular bone in CBCT images, leading to lower FD values that indicate greater motion unsharpness. The application of the CALM^®^ motion artifact reduction algorithm can mitigate this effect in some cases, restoring FD values closer to those of baseline scans without motion. However, the effectiveness of CALM^®^ varies depending on the site and degree of motion, and in some cases, it may affect the measurements even in the absence of motion. These findings highlight the importance of considering motion and the use of artifact reduction algorithms when interpreting FD analysis results in CBCT imaging. Further research is needed to optimize the use of such algorithms and to understand their impact on different sites and under varying degrees of motion.

## Figures and Tables

**Table 1:** FD values for Site 1 [Right Molars] including the Mean, Std. Deviation, Std. Error, and 95% Confidence Interval. It shows *p* values compared to Groups 1 and 2.

Site	Group	N	Mean	Std. Deviation	Std. Error	95% Confidence Interval for Mean		Minimum	Maximum	*p* Value in Comparison to Group 1	*p* Value in Comparison to Group 2
						Lower Bound	Upper Bound				
**Site 1** **[RM]**	1	10	1.166	0.029	0.009	1.145	1.187	1.122	1.206	-	0.975
	2	10	1.136	0.027	0.009	1.116	1.155	1.104	1.193	0.975	-
	3	10	1.133	0.059	0.019	1.090	1.175	1.041	1.222	0.959	1.000
	4	10	1.140	0.060	0.019	1.097	1.183	1.039	1.218	0.989	1.000
	5	10	1.156	0.047	0.015	1.123	1.190	1.071	1.215	1.000	0.997
	6	10	1.139	0.039	0.012	1.111	1.167	1.088	1.188	0.988	1.000
	7	10	1.044	0.137	0.043	0.946	1.142	0.785	1.241	0.005	0.082
	8	10	1.109	0.087	0.027	1.047	1.171	0.943	1.238	0.604	0.990
	Total	80	1.128	0.075	0.008	1.111	1.145	0.785	1.241		

**Table 2:** FD values for Site 2 [right premolars] including the Mean, Std. Deviation, Std. Error, and 95% Confidence Interval. It shows *p* values compared to Groups 1 and 2.

Site	Group	N	Mean	Std. Deviation	Std. Error	95% Confidence Interval for Mean		Minimum	Maximum	*p* Value in Comparison to Group 1	*p* Value in Comparison to Group 2
						Lower Bound	Upper Bound				
**Site 2** **RPM**	1	10	1.153	0.025	0.008	1.135	1.171	1.116	1.185	-	1.000
	2	10	1.136	0.025	0.008	1.118	1.154	1.093	1.161	1.000	-
	3	10	1.128	0.084	0.027	1.068	1.189	0.964	1.237	0.996	1.000
	4	10	1.170	0.022	0.007	1.153	1.186	1.142	1.208	1.000	0.976
	5	10	1.008	0.077	0.024	0.953	1.063	0.845	1.136	0.002	0.009
	6	10	1.008	0.103	0.033	0.934	1.082	0.792	1.167	0.002	0.009
	7	10	1.085	0.092	0.029	1.019	1.151	0.950	1.197	0.505	0.818
	8	10	0.994	0.115	0.036	0.912	1.077	0.823	1.199	<0.001	0.002
	Total	80	1.085	0.100	0.011	1.063	1.107	0.792	1.237		

**Table 3:** FD values for Site 3 [anterior] including the Mean, Std. Deviation, Std. Error, and 95% Confidence Interval. It shows *p* values compared to Groups 1 and 2.

Site	Group	N	Mean	Std. Deviation	Std. Error	95% Confidence Interval for Mean		Minimum	Maximum	*p* Value in Comparison to Group 1	*p* Value in Comparison to Group 2
						Lower Bound	Upper Bound				
**Site 3** **Anterior**	1	10	1.099	0.023	0.007	1.082	1.115	1.055	1.127	-	1.000
	2	10	1.092	0.031	0.010	1.070	1.115	1.039	1.140	1.000	-
	3	10	1.109	0.030	0.009	1.088	1.130	1.067	1.149	1.000	0.992
	4	10	1.113	0.022	0.007	1.097	1.129	1.084	1.146	0.997	0.975
	5	10	1.059	0.067	0.021	1.011	1.107	0.959	1.162	0.538	0.734
	6	10	1.044	0.063	0.020	0.999	1.089	0.924	1.105	0.152	0.277
	7	10	1.003	0.064	0.020	0.957	1.049	0.892	1.097	<0.001	0.001
	8	10	1.051	0.036	0.011	1.025	1.076	1.000	1.116	0.287	0.464
	Total	80	1.071	0.057	0.006	1.058	1.084	0.892	1.162		

**Table 4:** FD values for Site 4 [left premolars] including the Mean, Std. Deviation, Std. Error, and 95% Confidence Interval. It shows *p* values compared to Groups 1 and 2.

Site	Group	N	Mean	Std. Deviation	Std. Error	95% Confidence Interval for Mean		Minimum	Maximum	*p* Value in Comparison to Group 1	*p* Value in Comparison to Group 2
						Lower Bound	Upper Bound				
**Site 4** **LPM**	1	10	1.045	0.045	0.014	1.012	1.077	0.976	1.095	-	1.000
	2	10	1.054	0.051	0.016	1.018	1.090	0.977	1.122	1.000	-
	3	10	1.132	0.065	0.021	1.085	1.179	0.993	1.214	0.086	0.176
	4	10	1.126	0.100	0.032	1.054	1.198	0.989	1.259	0.138	0.263
	5	10	1.133	0.047	0.015	1.099	1.166	1.069	1.223	0.083	0.170
	6	10	1.159	0.044	0.014	1.128	1.191	1.073	1.215	0.007	0.017
	7	10	1.027	0.063	0.020	0.982	1.072	0.882	1.104	0.999	0.984
	8	10	1.100	0.096	0.030	1.031	1.168	0.938	1.209	0.604	0.799
	Total	80	1.097	0.079	0.009	1.079	1.114	0.882	1.259		

**Table 5:** FD values for Site 5 [left molar] including the Mean, Std. Deviation, Std. Error, and 95% Confidence Interval. It shows *p* values compared to Groups 1 and 2.

Site	Group	N	Mean	Std. Deviation	Std. Error	95% Confidence Interval for Mean		Minimum	Maximum	*p* Value in Comparison to Group 1	*p* Value in Comparison to Group 2
						Lower Bound	Upper Bound				
**Site 5** **LM**	1	10	1.045	0.162	0.051	0.929	1.161	0.882	1.292	-	0.040
	2	10	1.175	0.125	0.040	1.086	1.265	0.971	1.269	0.040	-
	3	10	1.026	0.094	0.030	0.959	1.093	0.949	1.265	1.000	0.010
	4	10	0.993	0.040	0.013	0.965	1.022	0.936	1.050	0.908	0.001
	5	10	0.986	0.048	0.015	0.952	1.021	0.920	1.052	0.838	<0.001
	6	10	1.003	0.063	0.020	0.958	1.048	0.896	1.086	0.968	0.002
	7	10	1.019	0.068	0.021	0.970	1.067	0.928	1.118	0.998	0.006
	8	10	0.984	0.056	0.018	0.945	1.024	0.917	1.051	0.813	<0.001
	Total	80	1.029	0.105	0.012	1.006	1.052	0.882	1.292		

**Table 6:** FD values for Site 5 [left molar] with additional scans and measurements, including the Mean, Std. Deviation, Std. Error, and 95% Confidence Interval. It shows *p* values compared to Groups 1 and 2.

Group	N	Mean	Std. Deviation	Std. Error	95% Confidence Interval for Mean		Minimum	Maximum	*p* Value in Comparison to Group 1	*p* Value in Comparison to Group 2
					Lower Bound	Upper Bound				
**1**	10	1.0604	0.15871	0.05019	0.9469	1.1739	0.86	1.29	-	0.997
**2**	10	1.0527	0.02951	0.00933	1.0316	1.0738	1.01	1.09	0.997	-
**3**	10	0.9808	0.03282	0.01038	0.9573	1.0043	0.94	1.04	0.187	0.265
**4**	10	0.9196	0.05350	0.01692	0.8813	0.9579	0.83	1.01	0.005	0.008
**Total**	80	1.0034	0.10154	0.01605	0.9709	1.0358	0.83	1.29		

## Data Availability

The data presented in this study are available on request from the corresponding author. The data are not publicly available due to privacy.
